# Feasibility of placenta-derived mesenchymal stem cells as a tool for studying pregnancy-related disorders

**DOI:** 10.1038/srep46220

**Published:** 2017-04-12

**Authors:** Naoki Fuchi, Kiyonori Miura, Hanako Doi, Tao-Sheng Li, Hideaki Masuzaki

**Affiliations:** 1 Department of Obstetrics and Gynaecology, Nagasaki University Graduate School of Medicine, Nagasaki, Japan; 2 Department of Stem Cell Biology, Atomic Bomb Disease Institute, Nagasaki University, Nagasaki, Japan

## Abstract

The cellular and molecular mechanisms responsible for pregnancy-related disorders remain unclear. We investigated the feasibility of using placenta-derived mesenchymal stem cells (MSCs) as a tool to study such pregnancy-related disorders. We isolated and expanded adequate numbers of cells with characteristic features of MSCs from the chorionic plate (CP-MSCs), chorionic villi (CV-MSCs), and decidua basalis (DB-MSCs) of human term placental tissues. All placenta-derived MSCs expressed pregnancy-associated C14MC microRNA (miRNA) (miR-323-3p). Interestingly, the placenta-specific C19MC miRNAs (miR-518b and miR517a) were clearly expressed in CP-MSCs and CV-MSCs of foetal origin, but were barely expressed in DB-MSCs of maternal origin. Furthermore, expression levels of placenta-specific C19MC miRNAs in CV-MSCs remained stable during the *ex vivo* expansion process and across different pregnancy phases (first trimester *versus* third trimester). High-efficiency siRNA transfection was confirmed in twice-passaged CV-MSCs with little toxicity, and microarray analysis was used to screen for miR-518b target genes. Placenta-derived MSCs, especially CV-MSCs, are a potential tool for investigating the role of placental miRNAs in pregnancy-related disorders.

The placenta plays an essential role in foetal development by providing nutrition and gas exchange to the foetus and by supporting immunological tolerance. Problems with the development and maintenance of the placenta can therefore result in pregnancy-related disorders, such as preeclampsia or foetal growth restriction. However, the mechanisms responsible for such pregnancy-related disorders remain poorly understood.

MicroRNAs (miRNAs) regulate various important physiological and pathological processes, including embryonic development^1^. The pregnancy-associated chromosome 14 miRNA cluster (C14MC) and chromosome 19 miRNA cluster (C19MC) miRNAs are predominantly expressed in human placental tissue during pregnancy and play a crucial role in placental development^2^,^3^. C19MC miRNAs have demonstrated an association with preeclampsia caused by abnormal development of placental vessels in early pregnancy^4^. Furthermore, trophoblast cells may release exosomes containing C19MC miRNAs, which enable foetoplacental–maternal communication by affecting both local and distant target tissues^3^,^5^. Pregnancy-associated miRNAs have been identified in the maternal circulation^6^, and we have previously reported that C19MC miRNAs in maternal plasma may serve as a useful biomarker for pregnancy-related disorders^7^,^8^,^9^,^10^,^11^.

Pregnancy-related disorders are thought to be associated with biological abnormalities of trophoblast cells. However, the use of primary trophoblast cells to study such disorders has been limited by their short life span and poor proliferation *in vitro*^12^. In contrast, mesenchymal stem cells (MSCs), which have been identified in most tissues of the body, including the umbilical cord and placenta^13^, demonstrate vigorous proliferative potential *in vitro*. Placenta-derived MSCs have therefore emerged as both an alternative source for regenerative medicine, and also as a tool for use in experimental studies. Interestingly, C19MC miRNAs were shown to be expressed in MSCs from first-trimester placenta^14^. Umbilical cord-derived MSCs were recently found to enhance the migration and proliferation of trophoblast cells^15^. Furthermore, preeclampsia-placenta-derived MSCs induced a preeclampsia-like phenotype in normal chorionic villi^16^, indicating the potential role of endogenous MSCs in placental development and maintenance. These results indicate the potential use of placenta-derived MSCs as a tool for investigating the mechanisms behind pregnancy-related disorders.

In this study, we aimed to expand MSCs from different parts of human term placentas including the chorionic plate (CP) and chorionic villi (CV) of foetal origin, and the decidua basalis (DB) of maternal origin (Fig. 1), and to examine the expression of pregnancy-associated miRNAs in MSCs primarily expanded from placental tissues. We also investigated the transfection efficiency of CP-MSCs and CV-MSCs from term placentas with small interfering RNAs (siRNAs), and miR-518b target genes were further screened for by microarray analysis. These preliminary results suggest that CV-MSCs are a potential tool for investigating the role of placental miRNAs in pregnancy-related disorders.

## Results

### Propagation and characterisation of placenta-derived MSCs

**Figure 1:**
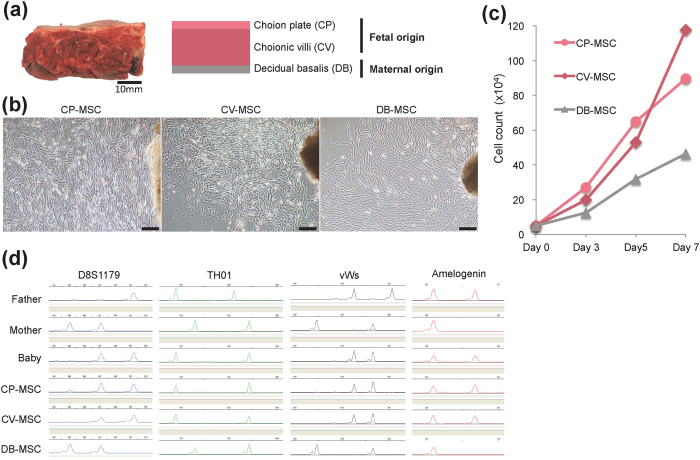
Propagation of mesenchymal stem cells (MSCs) derived from human term placental tissue. (**a**) Gross anatomical (left) and schematic (right) images of term placenta. Tissues from chorionic plate (CP), chorionic villi (CV), and decidua basalis (DB) were separated manually and collected for *ex vivo* expansion of MSCs. (**b**) Phase-contrast microscopic images showing outgrowth of fibroblast-like cells from explants of CP (CP-MSCs), CV (CV-MSCs), and DB (DB-MSCs) at about 10 days after initiation of cultures. Scale bars: 200 μm. (**c**) Growth kinetics of twice-passaged CP-MSCs, CV-MSCs, and DB-MSCs. (**d**) Representative electrophoretogram of microsatellite genotyping using representative short tandem repeat markers D8S1179, TH01, vWs, and amelogenin. CP-MSC and CV-MSC matched the baby (cord blood), while DB-MSC matched the mother. Amelogenin confirmed the presence of the X chromosome-specific allele alone for DB-MSC (mother), and both X and Y chromosome-specific alleles for CP-MSC and CV-MSC (baby).

We separated different parts (CP, CV, and DB) of human third-trimester placental tissues and cultured explants to generate MSCs (Fig. 1a). All cells that grew out from CP, CV, and DB tissues were morphologically fibroblast-like cells (Fig. 1b). The average population-doubling times of CP-MSCs, CV-MSCs, and DB-MSCs at passage two were 42.8 ± 6.3, 37.0 ± 1.4, and 68.1 ± 30.3 h, respectively (Fig. 1c). The origin of the MSCs was examined by microsatellite genotyping of the short tandem repeat (STR) markers D8S1179, TH01, vWs, and amelogenin (Fig. 1d), which revealed the biparental origins of CP-MSCs and CV-MSCs, compared with maternal-origin DB-MSCs.

**Figure 2:**
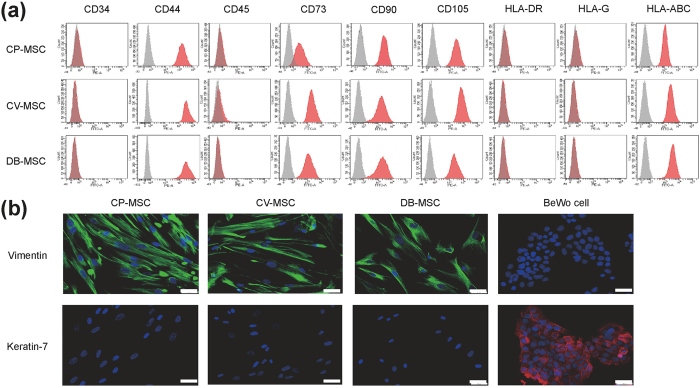
Characteristics of mesenchymal stem cells (MSCs) primarily expanded from human term placental tissue. Twice-passaged MSCs from chorionic plate (CP-MSCs), chorionic villi (CV-MSCs), and decidua basalis (DB-MSCs) were used for experiments. (**a**) Flow cytometry analysis showing expression profiles of cell surface markers on cells (grey areas indicated isotype negative controls). (**b**) Immunocytochemical images showing CP-MSCs, CV-MSCs, and DB-MSCs positively stained for the mesenchymal marker vimentin (*green*) but negatively stained for the pan-trophoblast marker keratin-7 (*red*). Nuclei were counterstained with DAPI (*blue*). BeWo trophoblast cells were used as control. Scale bars: 50 μm.

The phenotypes of the MSCs were examined by flow cytometry and immunocytochemistry (Fig. 2). Flow cytometry analysis showed that all CP-MSCs, CV-MSCs, and DB-MSCs expressed the MSC markers CD44, CD73, CD90, and CD105 but not the haematopoietic cell markers CD34 and CD45 (Fig. 2a). CP-MSCs, CV-MSCs, and DB-MSCs also expressed human leukocyte antigens (HLA)-A, B, and C (MHC class 1 cell surface receptors) but not HLA-DR (MHC class 2 cell surface receptor) (Fig. 2a), or HLA-G (MHC class 1 cell surface receptor, which is known to be expressed in extravillous trophoblasts) (Fig. 2a). According to immunocytochemistry analysis, all the MSCs primarily expanded from different parts of the placenta tissue were negatively stained for the pan-trophoblast-specific marker keratin 7 but positively stained for the mesenchymal marker vimentin (Fig. 2b).

### Placenta-derived MSCs positively expressed pregnancy-associated miRNAs

**Figure 3:**
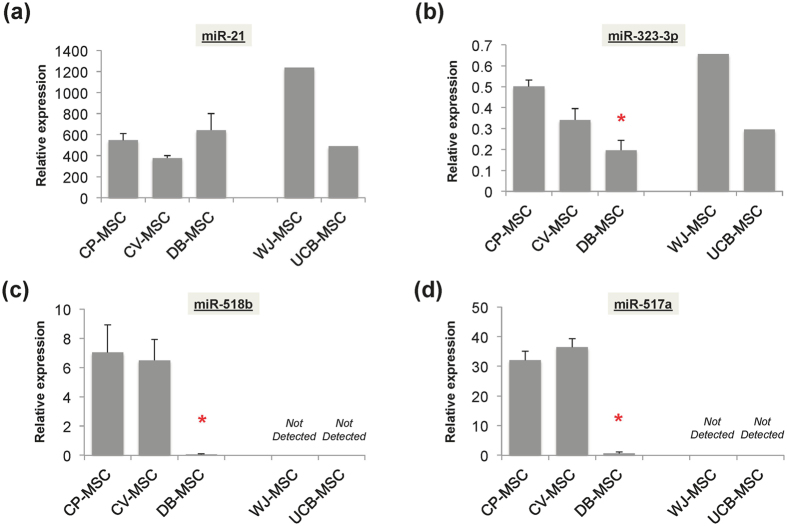
Expression levels of pregnancy-associated miRNAs. Twice-passaged mesenchymal stem cells (MSCs) from chorionic plate (CP-MSCs), chorionic villi (CV-MSCs), and decidua basalis (DB-MSCs) from human term placental tissue were used for experiments. (**a**) Expression of miR-21, a common miRNA widely found in various tissues/cells; (**b**) miR-323-3p, representative member of C14MC miRNA; and (**c**) miR-518b and (**d**) miR-517a, two representative members of C19MC miRNA were measured by qRT-PCR. Semi-quantitative data were presented as relative expression ratio after normalisation with U6 snRNA. *p < 0.05 *vs*. CP-MSCs and CV-MSCs.

The expression of miRNAs in placenta-derived MSCs was evaluated by quantitative reverse transcription polymerase chain reaction (qRT-PCR). miR-21, which is known to be widely expressed in various tissues/cells, was expressed at similar levels in CP-MSCs, CV-MSCs, and DB-MSCs (Fig. 3a), while expression of miR-323-3p, a representative C14MC miRNA, was significantly higher in CP-MSCs and CV-MSCs than in DB-MSCs (p < 0.001 *vs*. CP-MSCs and CV-MSCs) (Fig. 3b). Interestingly, the representative placenta-specific C19MC miRNAs miR-518b and miR-517a were only clearly expressed in CP-MSCs and CV-MSCs, and barely expressed in DB-MSCs (p < 0.001 *vs*. CP-MSCs and CV-MSCs) (Fig. 3c,d). Similarly, MSCs primarily expanded from umbilical cord blood (UCB-MSCs) and Wharton’s jelly tissue (WJ-MSCs) both expressed miR-323-3p (Fig. 3b) but not miR-518b and miR-517a (Fig. 3c,d).

**Figure 4:**
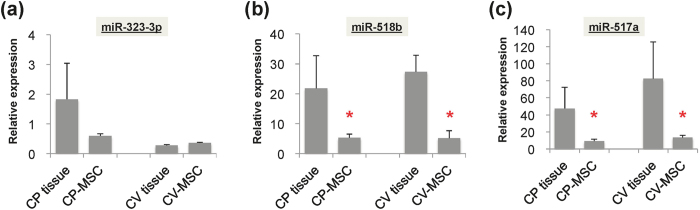
Expression levels of pregnancy-associated miRNAs in primarily expanded MSCs and their original placental tissues. (**a**) Expression levels of miR-323-3p, (**b**) miR-518b, and (**c**) miR-517a were measured by qRT-PCR analysis and represented as relative expression ratio after normalisation with U6 snRNA. Twice-passaged MSCs primarily expanded from chorionic plate (CP-MSCs) and chorionic villi (CV-MSCs) were compared with their original placental tissues. *p < 0.05 *vs*. placental tissues.

We also compared the expression levels of pregnancy-associated miRNAs between primarily expanded MSCs and their original placental tissues. The relative expression levels of miR-323-3p varied between primarily expanded MSCs and their original tissues (Fig. 4a), while expression levels of C19MC miRNAs (miR-518b and -517a) were significantly higher in CP and CV tissues than in twice-passaged CP-MSCs and CV-MSCs (p < 0.001 *vs*. CP tissues or CV tissues, respectively) (Fig. 4b,c).

### Expression of pregnancy-associated miRNAs in placental MSCs remained stable through different pregnancy phases and during *ex vivo* expansion

**Figure 5:**
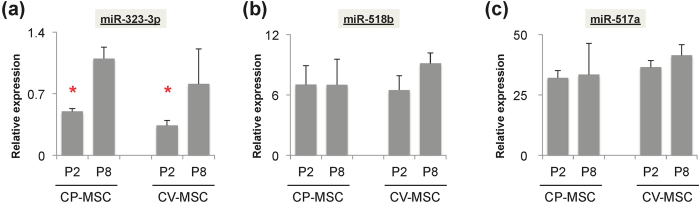
Changes in expression levels of pregnancy-associated miRNAs in mesenchymal stem cells (MSCs) during *ex vivo* expansion. (**a**) Expression levels of miR-323-3p, (**b**) miR-518b, and (**c**) miR-517a were measured by qRT-PCR and represented as relative expression ratios after normalisation with U6 snRNA. Twice-passaged (P2) MSCs from chorionic plate (CP-MSCs) and chorionic villi (CV-MSCs) were compared with later passages (P8). *p < 0.05 *vs*. P8.

As a potential tool for investigating the mechanisms responsible for pregnancy-related disorders, it is essential to understand the stability of pregnancy-associated miRNA expression levels in these placenta-derived MSCs. We therefore determined if the expression of pregnancy-associated miRNAs changed during the *ex vivo* expansion process. The expression of miR-323-3p in CP-MSCs and CV-MSCs was significantly higher in later (p8) compared with earlier passage (p2) cells (p < 0.001 *vs*. P8) (Fig. 5a), while levels of miR-518b and miR-517a remained relatively stable during *ex vivo* expansion (Fig. 5b,c).

**Figure 6:**
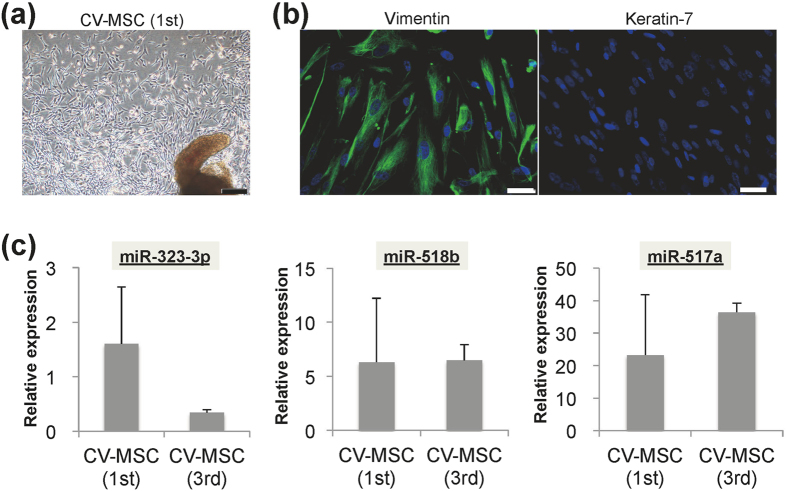
Mesenchymal stem cells (MSCs) primarily expanded from chorionic villi (CV-MSCs) from first-trimester placental tissues. (**a**) Representative phase-contrast microscopic image showing fibroblast-like cells growing out from chorionic villi from first-trimester placental tissues at 10 days after culture. Scale bars: 200 μm. (**b**) Immunocytochemical images showing expression of the mesenchymal marker vimentin (*green*) and pan-trophoblast marker keratin-7 (*red*) in twice-passaged CV-MSCs from first-trimester placenta. Nuclei are counterstained with DAPI (*blue*). Scale bars: 50 μm. (**c**) qRT-PCR analysis of expression of miR-323-3p, miR-518b, and miR-517a in twice-passaged CV-MSCs from first- and third-trimester placentas. Data are presented as relative expression ratio after normalisation with U6 snRNA.

We also primarily expanded and compared the properties of first- and third-trimester CV-MSCs, and showed that they had similar morphological features and cellular properties (Fig. 6a,b). However, first-trimester CV-MSCs showed relatively higher expression of miR-323-3p compared with third-trimester CV-MSCs, though the difference was not significant (Fig. 6c), while miR-518b and -517a levels were similar in cells from both trimesters (Fig. 6c).

**Figure 7:**
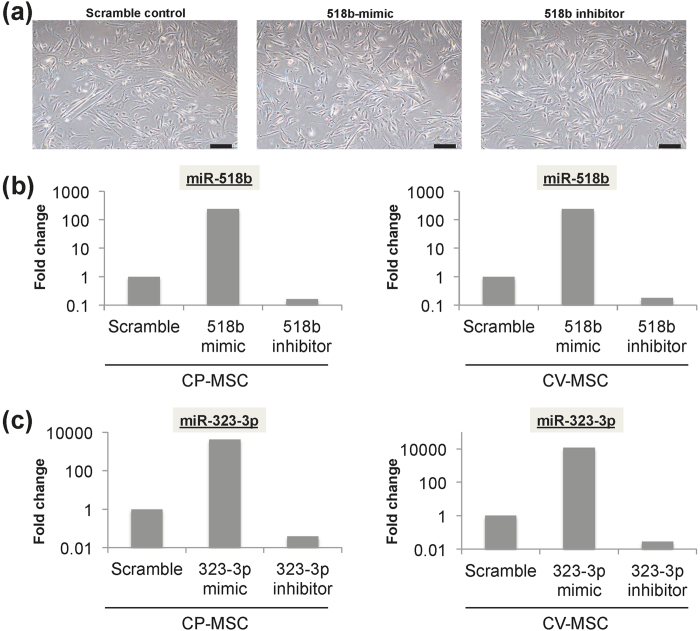
Efficiency of siRNA transfection of twice-passaged mesenchymal stem cells (MSCs) from chorionic plate (CP-MSCs) and chorionic villi (CV-MSCs) from term placentas. (**a**) Representative phase-contrast microscopic images showing similar morphological features of CV-MSCs at 48 h after siRNA transfection with scramble control, 518b-mimic, and 518b inhibitor. Scale bars: 200 μm. Fold changes in expression levels of miR-518b (**b**) and miR-323-3p (**c**) compared with scramble control in CP-MSCs and CV-MSCs.

We determined the effects of siRNA transfection in twice-passaged CP-MSCs and CV-MSCs derived from third-trimester placenta. siRNA transfection had little toxic effect (Fig. 7a). Furthermore, expression levels of miR-518b and miR-323-3p were effectively facilitated and suppressed by transfection with miRNA mimic and miRNA inhibitor, respectively (Fig. 7b).

### Screening for miR-518b target genes associated with pregnancy-related disorders

The main function of miRNAs is to regulate gene expression via antisense complimentarily to one or more messenger RNAs (mRNAs)^17^,^18^,^19^. We initially screened for miR-518b target genes by transfection of twice-passaged CV-MSCs with an miR-518b mimic. Microarray analysis indicated a number of genes that were up- or down-regulated by the miR-518b mimic. Among the 124 target genes down-regulated more than 2-fold (Supplementary Table S1), two genes (tyrosine hydroxylase: *TH* and hydroxy-delta-5-steroid dehydrogenase, 3 beta- and steroid delta-isomerase 1: *HSD3B1*) were previously demonstrated to be involved in the pathogenesis of preeclampsia^20^,^21^,^22^, and five genes (endothelin receptor type A: *EDNRA*, advanced glycosylation end product-specific receptor: *AGER*, wingless-type MMTV integration site family member 2: *WNT2*, complement component 9: *C9*, and transient receptor potential cation channel, subfamily M, member 2: *TRPM2*) play roles in preeclampsia with foetal growth restriction^23^,^24^,^25^,^26^,^27^,^28^,^29^,^30^,^31^,^32^ (Table 1). Likewise, among the 112 target genes up-regulated over 2-fold by the miR-518b mimic (Supplementary Table S2), four genes [hemopexin: *HPX*, serpin peptidase inhibitor, clade B (ovalbumin), member 2: *SERPINB2*, lipoprotein, Lp(a): *LPA*, and tumour necrosis factor superfamily, member 10: *TNFSF10*] show involvement in preeclampsia^35^,^36^,^37^,^38^, and two genes (CD69 molecule: *CD69* and stanniocalcin 1: *STC1*) are involved in preeclampsia with foetal growth restriction^33^,^34^,^39^ (Table 1).

## Discussion

The cytological characteristics of MSCs vary according to their origin^40^. Placenta-derived MSCs were recently reported to possess better immunoregulatory properties than umbilical cord-derived MSCs^41^. Furthermore, MSCs from the amnion, chorion, and umbilical cord of human placenta tissues have different gene expression profiles and differentiation capacities^42^, suggesting the existence of heterogeneity among MSCs originating from different tissues. However, the biological features of MSCs in placental tissues and their role in regulating placental development remain poorly understood. The present study aimed to investigate the feasibility of using placenta-derived MSCs as a tool to study the mechanisms responsible for pregnancy-related disorders.

We initially expanded MSCs from placenta tissues by seeding tissue explants from different parts of the placenta onto culture dishes. These explants produced adequate numbers of cells with high proliferative potency. Numerous methods have been used to isolate/expand MSCs from placental tissue, including enzymatic digestion of tissues to harvest MSCs as a single-cell suspension for further cell expansion^43^,^44^, or by seeding tissue fragments, as in the current study. Contamination between maternal- and foetal-origin MSCs remains a problem with expansion of MSCs from placental tissues^45^,^46^, but we were able to generate pure cells without contamination. The explant culture method also has the advantages of maintaining stemness and retaining identical cell properties over time^47^,^48^.

Considering the distinct role of pregnancy-associated miRNAs in placental development, we investigated the expression levels of the major clusters of pregnancy-associated miRNAs, C19MC and C14MC, in these MSCs primarily expanded from human placental tissues. C19MC miRNAs are placenta-specific miRNAs regulated by genomic imprinting, with only the paternally inherited allele being expressed in the placenta^49^,^50^. In contrast, C14MC miRNAs are expressed in both embryonic and placental tissues^51^,^52^, and are generally accepted as pregnancy-associated, rather than placenta-specific miRNAs^2^,^3^. C14MC miRNAs (miR-323-3p) were expressed in all the placenta-derived MSCs, including CP-MSCs, CV-MSCs, and DB-MSCs, and also in WJ-MSCs and UCB-MSCs. In contrast, the placenta-specific C19MC miRNAs (miR-518b and miR517a) were only clearly detected in CP-MSCs and CV-MSCs, with low or absent expression in DB-MSCs, WJ-MSCs, and UCB-MSCs.

In accord with a previous study^2^, expression levels of C14MC miRNAs were lower in third- compared with first-trimester CV-MSCs, and decreased with pregnancy progression. Interestingly, expression levels of the placenta-specific C19MC miRNAs were comparable in first- and third-trimester CV-MSCs, and remained stable during the *ex vivo* expansion process (within approximately 60 days). We also compared the expression of C19MC miRNAs between primarily expanded MSCs and their original tissues, and showed lower expression levels in CP-MSCs and CV-MSCs compared with the equivalent original tissues. Given that C19MC miRNAs are highly expressed in trophoblast cells^53^, it is not surprising that they were less enriched in placenta-derived MSCs compared with their original tissues.

Various miRNAs, especially pregnancy-associated miRNAs, have been implicated in pregnancy-related disorders, such as preeclampsia and foetal growth restriction^8^,^10^,^52^. MiRNAs have also frequently been used in overexpression or knockdown experiments of targeted genes to elucidate miRNA functions in the placenta. We confirmed the efficiency of siRNA transfection in these primarily expanded placental MSCs, with no obvious toxic effects. By transfection of CV-MSCs with the miR-518b mimic, we screened for potential miR-518b target genes by microarray analysis. miR-518b seems to control multiple target genes located on various chromosomes. Interestingly, some miR-518b target genes were previously demonstrated to associate with preeclampsia (e.g., *TH* and *HSD3B1* for down-regulated genes, and *HPX, SERPINB2, LPA* and *TNFSF10* for up-regulated genes)^20^,^21^,^22^,^35^,^36^,^37^,^38^ and with preeclampsia with foetal growth restriction (e.g., *EDNRA, AGER, WNT2, C9*, and *TRPM2* for down-regulated genes, and *CD69* and *STC1* for up-regulated genes)^23^,^24^,^25^,^26^,^27^,^28^,^29^,^30^,^31^,^32^,^33^,^34^,^39^. However, further experiments are needed to demonstrate a causal relationship between the expression of pregnancy-associated miRNAs and pregnancy-related disorders.

Placenta-derived MSCs are expected to be useful tools for studying pregnancy-related disorders because they are easily expanded *ex vivo*, they express specific pregnancy-associated miRNAs, and they show high transfection efficiencies for siRNAs. In this study, we only expanded MSCs from placental tissues from uncomplicated pregnancies. However, we also revealed different characteristics among CP-MSCs, CV-MSCs and DB-MSCs, supporting the idea of heterogeneity and tissue-specificity among placental MSCs for cell properties and proliferative capacities^13^,^42^,^44^,^54^. We have started to expand and characterize placental MSCs from abnormal conditions (e.g. preeclampsia and/or foetal growth restriction); however, there is much to learn to understand the functional roles and detailed mechanisms of action of pregnancy-associated miRNAs in pregnancy-related disorders.

In summary, we successfully expanded CP-MSCs, CV-MSCs, and DB-MSCs from placental tissue, resulting in cells with high proliferative potency and clear expression of pregnancy-associated miRNAs. The expression of placenta-specific C19MC miRNAs in CV-MSCs remained stable throughout different pregnancy phases and during *ex vivo* expansion. Placenta-derived MSCs, especially CV-MSCs, represent a potentially useful tool, not only for mechanistic understanding, but also for the treatment of pregnancy-related disorders^16^,^55^,^56^,^57^.

## Methods

### Ethics

This study was approved by the Institutional Review Boards for Ethical, Legal and Social Issues in Nagasaki University Graduate School of Biomedical Sciences (13052715). All samples were obtained after receiving written informed consent. The experiments were performed in accordance with the institutional and national guidelines.

### Primary isolation and expansion of human placental MSCs

We collected third-trimester (n = 3) placentas after elective caesarean section at 38–39 weeks of gestation, and first-trimester placentas (n = 3) after elective pregnancy termination at 8–10 weeks of gestation. We performed *ex vivo* expansion using explant methods, as described previously, with minor modifications^58^,^59^. Briefly, placental tissues were collected immediately after delivery and stored in Hank’s balanced salt solution (Life Technologies, Carlsbad, CA, USA) at 4 °C. After extensive washing in Dulbecco’s phosphate-buffered saline and mechanical removal of the amnion and blood vessels, third-trimester placental tissues were separated into CP, CV, and DB, but only CV was isolated from first-trimester placentas, because of the difficulty in discriminating between CP and DB. The tissues were cut into small pieces (1–2 mm) and cultured as explants on 6-cm culture dishes coated with 10 μg/ml human fibronectin (Corning, Corning, NY, USA). Within 1 week, fibroblast-like cells grew out from the tissue fragments, and became confluent at approximately 2 weeks. These cells were then collected using 0.25% trypsin-EDTA (Gibco, Waltham, MA, USA) and passaged for cell expansion. All cultures were incubated in a 5% CO_2_ incubator at 37 °C in Dulbecco’s modified Eagle’s medium (DMEM) (Wako, Osaka, Japan) supplemented with 10% foetal bovine serum (Hyclone Laboratories, Logan, UT, USA), 10 ng/ml human recombinant basic fibroblast growth factor (Wako), and 1% penicillin (100 U/ml)/streptomycin (100 U/ml) solution (Life Technologies).

### Culture of other cells

The BeWo trophoblast cell line, derived from a human gestational choriocarcinoma, was obtained from the Japanese Collection of Research Bioresources Cell Bank (Osaka, Japan) and maintained in Ham’s F-12 medium (Wako) supplemented with 15% foetal bovine serum and 1% penicillin/streptomycin.

UCB-MSCs and WJ-MSCs used as other-placental site-derived MSC models originated from the foetus, but not placenta, were kindly gifted by Dr. Doi^32^ and maintained in DMEM supplemented with 10% foetal bovine serum and 1% penicillin/streptomycin. Cells were cultured in a 5% CO_2_ incubator at 37 °C.

### Cell growth assay

Twice-passaged CP-MSCs, CV-MSCs, and DB-MSCs were seeded onto 6-well plates at a density of 5.2 × 10^3^ cells/cm^2^. The cells were then collected as single-cell suspensions at 3, 5, and 7 days, respectively. The total numbers of collected cells were counted with a haemocytometer to evaluate cell proliferation.

### Genotyping of STR polymorphisms

Genomic DNA was extracted using a QIAamp DNA Mini Kit (Qiagen, Hilden, Germany) from cultured MSCs, umbilical cord (baby) and parent peripheral blood samples. STR loci were analysed using the Powerplex 16 system (Promega, Madison, WI, USA) using an ABI PRISM3500 Genetic Analyzer (Applied Biosystems, Foster City, CA, USA) and GeneMapper software (Applied Biosystems), following the manufacturer’s instructions.

### Flow cytometry

Cells were harvested with 0.25% trypsin-EDTA. After washing with phosphate-buffered saline, the cells were then incubated with mouse monoclonal antibodies against CD34-fluorescein isothiocyanate (FITC) (4H11), CD44-phycoerythrin (PE) (IM7), CD45-PE (HI30), CD73-FITC (AD2), CD90-FITC (eBio5E10), CD105-PE (SN6), HLA-DR-PE (L243), HLA-G-PE (87G), and HLA-ABC-FITC (W6/32), respectively (eBioscience, San Diego, CA, USA). The respective isotopes were used as a negative control. Flow cytometry analysis was performed using an LSRFortessa (Becton Dickinson, Franklin Lakes, NJ, USA) and the acquired data were analysed using Cell Quest software (Becton Dickinson).

### Immunocytochemistry

Cells were fixed in 4% paraformaldehyde and blocked in 0.3% Triton X-100 and 1% bovine serum albumin, and then incubated with primary antibodies against vimentin (D21H3) and keratin 7 (D1E4) (Cell Signaling Technology, Danvers, MA, USA). Appropriate second antibodies conjugated with Alexa fluorochromes were used to detect positive staining. The nuclei were stained with 4′, 6-diamidino-2-phenylindole (DAPI), and positively stained cells were visualised under a fluorescence microscope.

### qRT-PCR

Total RNAs containing small RNA molecules were collected using mirVana miRNA Isolation Kit (Ambion, Waltham, MA, USA), according to the manufacturer’s instructions. Total RNAs were extracted from MSCs and from the original placental tissues for comparison. Total RNA concentrations were measured using NanoDrop 2000 (Thermo Fisher Scientific, Waltham, MA, USA). A total of 100 ng RNA was used for the following the steps. Five specific primers and TaqMan probes for test and control miRNAs (C19MC miRNAs miR-518b (assay ID 001156) and miR-517a (assay ID 002402); C14MC miRNA 323-3p (assay ID 002227); miR-21 (assay ID 000397); and U6 snRNA (assay ID 001973)) were used for TaqMan MicroRNA Assays (Applied Biosystems). Absolute qRT-PCR of miRNAs was performed as described previously^7^,^8^,^9^. For each miRNA assay, a calibration curve was prepared by 10-fold serial dilution of single-stranded cDNA oligonucleotides corresponding to each miRNA sequence from 1.0 × 10^2^ to 1.0 × 10^8^ copies/ml. Each sample and each calibration dilution were analysed in triplicate. The lower limit of detection for each assay was 300 RNA copies/ml^7^,^8^,^9^. Each batch of amplifications included three water blanks as negative controls for each of the reverse transcription and PCR steps. All the data were collected and analysed using a LightCycler^®^ 480 real-time PCR system (Roche, Basel, Switzerland). Expression levels were represented as relative ratios using U6 snRNA as an endogenous control for normalisation.

### Transfection of siRNA

Cells were transfected with synthetic miRNA mimic and miRNA inhibitor of hsa-miR-518b (assay ID MC12660 and MH12660) and -323-3p (assay ID MC12418 and MH12418) (Applied Biosystems), or with scramble controls (Ambion), using Lipofectamine 3000 reagent (Invitrogen, Carlsbad, CA, USA), according to the manufacturer’s instructions. A total of 30 nM of siRNA duplex was used for each transfection. Transfection efficiency was assessed by qRT-PCR.

### Microarray analysis

Total RNAs were collected from the control MSCs (untreated twice-passaged CV-MSCs) and twice-passaged CV-MSCs 72 h after transfection with an miRNA mimic of miR-518b. The integrity of total RNAs was estimated using a 2100 Bioanalyzer (Agilent Technologies, Santa Clara, CA, USA). Total RNAs (50 ng) of each MSC were labelled using a Low Input Quick Amp Labeling Kit (Agilent Technologies). miR-518b target genes were identified using a SuperPrint G3 human Microarray 8 × 60 ver. 3 (Agilent Technologies). The resulting intensity was normalized to the 75 percentile shift and the processed data was filtered for over 2-fold up- or down-regulation compared with control MSCs by GeneSpring Gx software ver. 13 (Agilent Technologies). Registered genes in the HUGO (Human Genome Organization) Gene Nomenclature Committee database (http://www.genenames.org/) are listed as gene symbols in Supplemental Tables 1 and 2.

### Statistical analyses

Data were shown as the mean ± standard error. Statistical significance was determined using Mann–Whitney *U* tests (SPSS ver. 23, IBM, Armonk, NY, USA). A p value < 0.05 was accepted as statistically significant.

## Additional Information

**How to cite this article:** Fuchi, N. *et al*. Feasibility of placenta-derived mesenchymal stem cells as a tool for studying pregnancy-related disorders. *Sci. Rep.* **7**, 46220; doi: 10.1038/srep46220 (2017).

**Publisher's note:** Springer Nature remains neutral with regard to jurisdictional claims in published maps and institutional affiliations.

## Supplementary Material

Supplementary Tables S1 and S2

## Figures and Tables

**Table 1:** Representative miR-518b target genes that were previously reported to associate with pregnancy-related disorders.

Gene symbol	Gene name	Fold change	Pregnancy-related disorders	References
**Down**-**regulated target genes**				
*TH*	Tyrosine hydroxylase	2.964	PE	20
*HSD3B1*	Hydroxy-delta-5-steroid dehydrogenase, 3 beta- and steroid delta-isomerase 1	2.572	PE	21,22
*EDNRA*	Endothelin receptor type A	2.639	PE, FGR	23,24
*AGER*	Advanced glycosylation end product-specific receptor	2.220	PE, FGR	25,26
*WNT2*	Wingless-type MMTV integration site family member 2	2.116	PE, FGR	27, 28, 29
*C9*	Complement component 9	2.073	PE, FGR	30
*TRPM2*	Transient receptor potential cation channel, subfamily M, member 2	2.042	PE, FGR	31,32
**Up**-**regulated target genes**				
*CD69*	CD69 molecule	3.179	PE, FGR	33,34
*HPX*	Hemopexin	2.721	PE	35
*SERPINB2*	Serpin peptidase inhibitor, clade B (ovalbumin), member 2	2.248	PE	36
*LPA*	Lipoprotein, Lp(a)	2.201	PE	37
*TNFSF10*	Tumour necrosis factor (ligand) superfamily, member 10	2.113	PE	38
*STC1*	Stanniocalcin 1	2.039	PE, FGR	39

PE: preeclampsia, FGR: foetal growth restriction.
