# Corrigendum: Feasibility of placenta-derived mesenchymal stem cells as a tool for studying pregnancy-related disorders

**DOI:** 10.1038/srep46922

**Published:** 2017-12-22

**Authors:** Naoki Fuchi, Kiyonori Miura, Hanako Doi, Tao-Sheng Li, Hideaki Masuzaki

Scientific Reports 7: Article number: 46220; 10.1038/srep46220 published online: 04 12 2017; updated: 12 22 2017.

The original version of this Article contained an error in Affiliation 1 which was incorrectly listed as ‘Department of Obstetrics and Gynaecology, Nagasaki University Graduate School of Medicine, Nagasaki, Japan’. The correct affiliation is listed below:

Department of Obstetrics and Gynaecology, Nagasaki University Graduate School of Biomedical Sciences, Nagasaki, Japan.

These errors have now been corrected in the PDF and HTML versions of the Article, and in the accompanying Supplementary Information file.

