# Supplementary material for: Feasibility of placenta-derived mesenchymal stem cells as a tool for studying pregnancy-related disorders
Source: Sci Rep. 2017 Apr 12;7:46220. doi: 10.1038/srep46220 (PMC5388876; doi:10.1038/srep46220)
Supplement: Supplementary Tables S1 and S2 [file srep46220-s1.pdf]

Supplementary Information

Feasibility of placenta-derived mesenchymal stem cells as a tool for studying pregnancy-related disorders

Naoki Fuchi[1,2], Kiyonori Miura[1,*], Hanako Doi[2], Tao-Sheng Li[2] and Hideaki Masuzaki[1]

[1]Department of Obstetrics and Gynaecology, Nagasaki University Graduate School of Medicine, Nagasaki, Japan

[2]Department of Stem Cell Biology, Atomic Bomb Disease Institute, Nagasaki University, Nagasaki, Japan

[*]**Correspondence to:**

Dr. Kiyonori Miura, Department of Obstetrics and Gynaecology, Nagasaki University Graduate School of Biomedical Sciences, 1-7-1 Sakamoto, Nagasaki 852-8501, Japan.

Tel.: +81-95-819-7363; Fax: +81-95-819-7365; E-mail: kiyonori@nagasaki-u.ac.jp

Supplementary Table S1. List of genes down-regulated over 2-fold by miR-518b mimic.

Gene symbol	Gene name	Location	Fold change
ADPGK-AS1	ADPGK antisense RNA 1	15q24.1	9.4406
RELN	reelin	7q22.1	9.3928
SPANXN4	SPANX family, member N4	Xq27.3	8.3370
KRTAP19-4	keratin associated protein 19-4	21q22.11	7.8214
HSD17B3	hydroxysteroid (17-beta) dehydrogenase 3	9q22.32	7.4708
LINC00917	long intergenic non-protein coding RNA 917	16q24.1	7.0429
ONECUT1	one cut homeobox 1	15q21.3	5.8116
MAGEB16	melanoma antigen family B, 16	Xp21.1	5.5108
IL20	interleukin 20	1q32.1	5.3073
CMAHP	cytidine monophospho-N-acetylneuraminic acid hydroxylase, pseudogene	6p22.3	5.2815
CAPSL	calcyphosine-like	5p13.2	5.2388
EFCAB1	EF-hand calcium binding domain 1	8q11.21	5.0843
OR6C6	olfactory receptor, family 6, subfamily C, member 6	12q13.2	4.9495
KCTD14	potassium channel tetramerization domain containing 14	11q14.1	4.9125
PDXK	pyridoxal (pyridoxine, vitamin B6) kinase	21q22.3	4.7193
ATG9B	autophagy related 9B	7q36.1	4.7060
DEFB136	defensin, beta 136	8p23.1	4.6641
SDK1	sidekick cell adhesion molecule 1	7p22.2	4.6099
PTOV1-AS1	PTOV1 antisense RNA 1	19q13.33	4.5673
KANSL1L	KAT8 regulatory NSL complex subunit 1-like	2q34	4.3124
USHBP1	Usher syndrome 1C binding protein 1	19p13.11	4.2736
KDM4D	lysine (K)-specific demethylase 4D	11q21	4.1256
GNRHR2	gonadotropin-releasing hormone (type 2) receptor 2, pseudogene	1q21.1	3.9931
LRRC38	leucine rich repeat containing 38	1p36.21	3.9415
CROCC	ciliary rootlet coiled-coil, rootletin	1p36.13	3.8701
KLK5	kallikrein-related peptidase 5	19q13.41	3.7649
UPB1	ureidopropionase, beta	22q11.23	3.6957
GRID1	glutamate receptor, ionotropic, delta 1	10q23.1	3.5212
CLLU1OS	chronic lymphocytic leukemia up-regulated 1 opposite strand	12q22	3.5138
BMP7	bone morphogenetic protein 7	20q13.31	3.3608
CCDC114	coiled-coil domain containing 114	19q13.33	3.2935
SAP30L-AS1	SAP30L antisense RNA 1 (head to head)	5q33.2	3.2777
JAM2	junctional adhesion molecule 2	21q21.3	3.2380
C16orf97	chromosome 16 open reading frame 97	16q12.1	3.2258
ANKRD20A2	ankyrin repeat domain 20 family, member A2	9p12	3.1160
AIFM2	apoptosis-inducing factor, mitochondrion-associated, 2	10q22.1	3.1061
IQCF2	IQ motif containing F2	3p21.2	3.1005
PPP1R37	protein phosphatase 1, regulatory subunit 37	19q13.32	3.0400
A2M	alpha-2-macroglobulin	12p13.31	2.9937
TH	tyrosine hydroxylase	11p15.5	2.9643
MAPT	microtubule-associated protein tau	17q21.31	2.8587
CDKN2B-AS1	CDKN2B antisense RNA 1	9p21.3	2.8158
DNAH12	dynein, axonemal, heavy chain 12	3p14.3	2.7903
TRIM49	tripartite motif containing 49	11q14.3	2.7782
FOXJ1	forkhead box J1	17q25.1	2.7513
ORC1	origin recognition complex, subunit 1	1p32.3	2.7511
RAB17	RAB17, member RAS oncogene family	2q37.3	2.7108
TECPR2	tectonin beta-propeller repeat containing 2	14q32.31	2.6690

EDNRA	endothelin receptor type A	4q31.22	2.6394
SCRG1	stimulator of chondrogenesis 1	4q34.1	2.6215
COX4I2	cytochrome c oxidase subunit IV isoform 2 (lung)	20q11.21	2.6059
L2HGDH	L-2-hydroxyglutarate dehydrogenase	14q21.3	2.5864
CEP164	centrosomal protein 164kDa	11q23.3	2.5824
HSD3B1	hydroxy-delta-5-steroid dehydrogenase, 3 beta- and steroid delta-isomerase 1	1p12	2.5720
MUC3A	mucin 3A, cell surface associated	7q22.1	2.5648
SOX10	SRY (sex determining region Y)-box 10	22q13.1	2.5465
KCTD21-AS1	KCTD21 antisense RNA 1	11q14.1	2.5302
LRRC15	leucine rich repeat containing 15	3q29	2.5122
WFDC1	WAP four-disulfide core domain 1	16q24.1	2.4971
MX1	MX dynamin-like GTPase 1	21q22.3	2.4932
PTPRG-AS1	PTPRG antisense RNA 1	3p14.2	2.4875
C1QTNF2	C1q and tumor necrosis factor related protein 2	5q33.3	2.4874
MGAT3	mannosyl (beta-1,4-)-glycoprotein beta-1,4-N-acetylglucosaminyltransferase	22q13.1	2.4619
TNFRSF9	tumor necrosis factor receptor superfamily, member 9	1p36.23	2.4344
INMT	indolethylamine N-methyltransferase	7p14.3	2.4323
CCDC138	coiled-coil domain containing 138	2q12.3	2.4059
CTAG1A	cancer/testis antigen 1A	Xq28	2.3611
LBH	limb bud and heart development	2p23.1	2.3559
STRA6	stimulated by retinoic acid 6	15q24.1	2.3487
SLC14A1	solute carrier family 14 (urea transporter), member 1 (Kidd blood group)	18q12.3	2.3187
SLC25A5-AS1	SLC25A5 antisense RNA 1	Xq24	2.3111
OR51D1	olfactory receptor, family 51, subfamily D, member 1	11p15.4	2.2960
ABCB9	ATP-binding cassette, sub-family B (MDR/TAP), member 9	12q24.31	2.2946
TNNT2	troponin T type 2 (cardiac)	1q32.1	2.2944
SYNE2	spectrin repeat containing, nuclear envelope 2	14q23.2	2.2809
ERVK13-1	endogenous retrovirus group K13, member 1	16p13.3	2.2638
HAPLN4	hyaluronan and proteoglycan link protein 4	19p13.11	2.2529
LINC01036	long intergenic non-protein coding RNA 1036	1q31.1	2.2517
TMSB15A	thymosin beta 15a	Xq22.1	2.2462
GUCY1B3	guanylate cyclase 1, soluble, beta 3	4q32.1	2.2448
B3GALT2	UDP-Gal:betaGlcNAc beta 1,3-galactosyltransferase, polypeptide 2	1q31.2	2.2301
TEX22	testis expressed 22	14q32.33	2.2250
AGER	advanced glycosylation end product-specific receptor	6p21.32	2.2201
EXTL1	exostosin-like glycosyltransferase 1	1p36.11	2.2187
CEP44	centrosomal protein 44kDa	4q34.1	2.2082
CBFA2T3	core-binding factor, runt domain, alpha subunit 2; translocated to, 3	16q24.3	2.2026
GORAB	golgin, RAB6-interacting	1q24.2	2.1934
LINC00593	long intergenic non-protein coding RNA 593	15q23	2.1799
SNORD117	small nucleolar RNA, C/D box 117	6p21.33	2.1734
TRIM62	tripartite motif containing 62	1p35.1	2.1683
CGREF1	cell growth regulator with EF-hand domain 1	2p23.3	2.1641
SNORA30	small nucleolar RNA, H/ACA box 30	16p11.2	2.1623
SORBS1	sorbin and SH3 domain containing 1	10q24.1	2.1570
DACH1	dachshund family transcription factor 1	13q21.33	2.1567
ZNF3	zinc finger protein 3	7q22.1	2.1520
C8orf87	chromosome 8 open reading frame 87	8q22.1	2.1504
HIST1H4A	histone cluster 1, H4a	6p22.2	2.1464
CACNA1B	calcium channel, voltage-dependent, N type, alpha 1B subunit	9q34.3	2.1394
E2F2	E2F transcription factor 2	1p36.12	2.1384

IRG1	immunoresponsive 1 homolog (mouse)	13q22.3	2.1371
ARHGAP26	Rho GTPase activating protein 26	5q31.3	2.1239
LCE2A	late cornified envelope 2A	1q21.3	2.1238
WNT2	wingless-type MMTV integration site family member 2	7q31.2	2.1156
DGCR5	DiGeorge syndrome critical region gene 5 (non-protein coding)	22q11.21	2.1112
ABCG5	ATP-binding cassette, sub-family G (WHITE), member 5	2p21	2.1108
WDR76	WD repeat domain 76	15q15.3	2.1107
LCORL	ligand dependent nuclear receptor corepressor-like	4p15.31	2.1024
CLSPN	claspin	1p34.3	2.1019
LINC01011	long intergenic non-protein coding RNA 1011	6p25.2	2.0964
KRTAP12-1	keratin associated protein 12-1	21q22.3	2.0846
HMX1	H6 family homeobox 1	4p16.1	2.0839
LINC00469	long intergenic non-protein coding RNA 469	17q25.1	2.0832
HELZ2	helicase with zinc finger 2, transcriptional coactivator	20q13.33	2.0798
C9	complement component 9	5p13.1	2.0730
GJD3	gap junction protein, delta 3, 31.9kDa	17q21.2	2.0698
LINC00652	long intergenic non-protein coding RNA 652	20p11.23	2.0596
HIST1H2AG	histone cluster 1, H2ag	6p22.1	2.0576
TRPM2	transient receptor potential cation channel, subfamily M, member 2	21q22.3	2.0420
OR9A2	olfactory receptor, family 9, subfamily A, member 2	7q34	2.0416
IFI44L	interferon-induced protein 44-like	1p31.1	2.0294
ITGB4	integrin, beta 4	17q25.1	2.0169
LINC00642	long intergenic non-protein coding RNA 642	14q32.11	2.0147
PPIH	peptidylprolyl isomerase H (cyclophilin H)	1p34.2	2.0125
ABCG4	ATP-binding cassette, sub-family G (WHITE), member 4	11q23.3	2.0105

Supplementary Table S2. List of genes up-regulated over 2-fold by miR-518b mimic.

Gene symbol	Gene name	Location	Fold change
CACNA1C-AS2	CACNA1C antisense RNA 2	12p13.33	23.545
LINC00905	long intergenic non-protein coding RNA 905	19p13.12	17.224
LINC00982	long intergenic non-protein coding RNA 982	1p36.32	14.273
NLRC4	NLR family, CARD domain containing 4	2p22.3	10.581
GJB3	gap junction protein, beta 3, 31kDa	1p34.3	9.169
SLC6A20	solute carrier family 6 (proline IMINO transporter), member 20	3p21.31	7.078
CHST6	carbohydrate (N-acetylglucosamine 6-O) sulfotransferase 6	16q23.1	6.459
ORAOV1	oral cancer overexpressed 1	11q13.3	6.269
ANTXRLP1	anthrax toxin receptor-like pseudogene 1	10q11.22	5.597
ZNF750	zinc finger protein 750	17q25.3	5.239
LZTS1	leucine zipper, putative tumor suppressor 1	8p21.3	4.910
XIRP2	xin actin-binding repeat containing 2	2q24.3	4.870
OR52E8	olfactory receptor, family 52, subfamily E, member 8	11p15.4	4.835
OR7A17	olfactory receptor, family 7, subfamily A, member 17	19p13.12	4.594
REG3A	regenerating islet-derived 3 alpha	2p12	4.232
RXFP4	relaxin/insulin-like family peptide receptor 4	1q22	3.848
KRTAP22-1	keratin associated protein 22-1	21q22.11	3.832
CD28	CD28 molecule	2q33.2	3.802
USHBP1	Usher syndrome 1C binding protein 1	19p13.11	3.763
TDRKH	tudor and KH domain containing	1q21.3	3.611
FADS6	fatty acid desaturase 6	17q25.1	3.501
C2orf66	chromosome 2 open reading frame 66	2q33.1	3.493
ZCCHC11	zinc finger, CCHC domain containing 11	1p32.3	3.447
LCE3C	late cornified envelope 3C	1q21.3	3.442
INTS6	integrator complex subunit 6	13q14.3	3.431
FCRL2	Fc receptor-like 2	1q23.1	3.396
SNORD82	small nucleolar RNA, C/D box 82	2q37.1	3.392
SYTL4	synaptotagmin-like 4	Xq22.1	3.336
FRMD4B	FERM domain containing 4B	3p14.1	3.211
CD69	CD69 molecule	12p13.31	3.179
OR2AG1	olfactory receptor, family 2, subfamily AG, member 1 (gene/pseudogene)	11p15.4	3.171
C1orf159	chromosome 1 open reading frame 159	1p36.33	3.082
TLE1	transducin-like enhancer of split 1 (E(sp1) homolog, Drosophila)	9q21.32	3.070
C16orf97	chromosome 16 open reading frame 97	16q12.1	3.021
OXR1	oxidation resistance 1	8q23.1	2.912
DMKN	dermokine	19q13.12	2.908
DOC2B	double C2-like domains, beta	17p13.3	2.899
LINC00160	long intergenic non-protein coding RNA 160	21q22.12	2.889
GBA3	glucosidase, beta, acid 3 (gene/pseudogene)	4p15.2	2.875
IQCF6	IQ motif containing F6	3p21.2	2.850
KLHDC7A	kelch domain containing 7A	1p36.13	2.830
LINC00520	long intergenic non-protein coding RNA 520	14q22.3	2.775
PTCHD4	patched domain containing 4	6p12.3	2.755
OLIG3	oligodendrocyte transcription factor 3	6q23.3	2.723
HPX	hemopexin	11p15.4	2.721
SLAMF7	SLAM family member 7	1q23.3	2.705
TMEM5-AS1	TMEM5 antisense RNA 1	12q14.2	2.629
LGI4	leucine-rich repeat LGI family, member 4	19q13.12	2.612

ADAM32	ADAM metallopeptidase domain 32	8p11.22	2.610
EGFEM1P	EGF-like and EMI domain containing 1, pseudogene	3q26.2	2.548
NRL	neural retina leucine zipper	14q11.2	2.508
PSG8	pregnancy specific beta-1-glycoprotein 8	19q13.2	2.497
CDH26	cadherin 26	20q13.33	2.493
NOVA2	neuro-oncological ventral antigen 2	19q13.32	2.484
ZFHX4-AS1	ZFHX4 antisense RNA 1	8q21.11	2.474
NCR2	natural cytotoxicity triggering receptor 2	6p21.1	2.473
FNDC3A	fibronectin type III domain containing 3A	13q14.2	2.443
XKR4	XK, Kell blood group complex subunit-related family, member 4	8q12.1	2.429
SERPINB11	serpin peptidase inhibitor, clade B (ovalbumin), member 11 (gene/pseudogene)	18q21.33	2.393
C17orf78	chromosome 17 open reading frame 78	17q12	2.364
EZR-AS1	EZR antisense RNA 1	6q25.3	2.355
SCN3B	sodium channel, voltage gated, type III beta subunit	11q24.1	2.354
NT5DC4	5'-nucleotidase domain containing 4	2q13	2.334
C19orf73	chromosome 19 open reading frame 73	19q13.33	2.327
VPS37A	vacuolar protein sorting 37 homolog A (S. cerevisiae)	8p22	2.316
RPGR	retinitis pigmentosa GTPase regulator	Xp11.4	2.297
OR5AS1	olfactory receptor, family 5, subfamily AS, member 1	11q12.1	2.295
HLA-DPB2	major histocompatibility complex, class II, DP beta 2 (pseudogene)	6p21.32	2.289
C17orf104	chromosome 17 open reading frame 104	17q21.31	2.272
KMT2E-AS1	KMT2E antisense RNA 1 (head to head)	7q22.3	2.268
KCTD21-AS1	KCTD21 antisense RNA 1	11q14.1	2.262
SERPINB2	serpin peptidase inhibitor, clade B (ovalbumin), member 2	18q21.33	2.248
MIR155HG	MIR155 host gene (non-protein coding)	21q21.3	2.244
PTGER3	prostaglandin E receptor 3 (subtype EP3)	1p31.1	2.220
NPHS1	nephrosis 1, congenital, Finnish type (nephrin)	19q13.12	2.215
WDR63	WD repeat domain 63	1p22.3	2.215
PLA2G2A	phospholipase A2, group IIA (platelets, synovial fluid)	1p36.13	2.209
CDH10	cadherin 10, type 2 (T2-cadherin)	5p14.2	2.208
SNRK-AS1	SNRK antisense RNA 1	3p22.1	2.206
BZRAP1-AS1	BZRAP1 antisense RNA 1	17q22	2.204
LPA	lipoprotein, Lp(a)	6q25.3	2.201
LRRC71	leucine rich repeat containing 71	1q23.1	2.194
SLC5A10	solute carrier family 5 (sodium/sugar cotransporter), member 10	17p11.2	2.194
RGS16	regulator of G-protein signaling 16	1q25.3	2.191
TMEM161B-AS1	TMEM161B antisense RNA 1	5q14.3	2.190
IFNAR2	interferon (alpha, beta and omega) receptor 2	21q22.11	2.189
PCDHGB6	protocadherin gamma subfamily B, 6	5q31.3	2.164
NRXN1	neurexin 1	2p16.3	2.163
LINC00689	long intergenic non-protein coding RNA 689	7q36.3	2.148
MCTP1	multiple C2 domains, transmembrane 1	5q15	2.128
C1orf115	chromosome 1 open reading frame 115	1q41	2.116
ZNF749	zinc finger protein 749	19q13.43	2.114
SCD5	stearoyl-CoA desaturase 5	4q21.22	2.113
TNFSF10	tumor necrosis factor (ligand) superfamily, member 10	3q26.31	2.113
CASC2	cancer susceptibility candidate 2 (non-protein coding)	10q26.11	2.107
KRT79	keratin 79, type II	12q13.13	2.082
G0S2	G0/G1 switch 2	1q32.2	2.079
DNM1	dynamin 1	9q34.11	2.062
LINC00672	long intergenic non-protein coding RNA 672	17q12	2.054

BMP1	bone morphogenetic protein 1	8p21.3	2.054
C15orf48	chromosome 15 open reading frame 48	15q21.1	2.052
CD177	CD177 molecule	19q13.31	2.051
DNASE1	deoxyribonuclease I	16p13.3	2.050
UCA1	urothelial cancer associated 1 (non-protein coding)	19p13.12	2.040
STC1	stanniocalcin 1	8p21.2	2.039
C6orf164	chromosome 6 open reading frame 164	6q15	2.038
RNF32	ring finger protein 32	7q36.3	2.037
GPR179	G protein-coupled receptor 179	17q12	2.032
MORN1	MORN repeat containing 1	1p36.32	2.016
NYNRIN	NYN domain and retroviral integrase containing	14q12	2.015
PAG1	phosphoprotein membrane anchor with glycosphingolipid microdomains 1	8q21.13	2.014
C4orf36	chromosome 4 open reading frame 36	4q21.3	2.013
